# 

**Published:** 1846-05

**Authors:** 


### WESTERN JOURNAL

OF

### MEDICINE AND SURGERY.

### EDITORS.

---

DANIEL DRAKE, M.D.,

PROFESSOR OF PATHOLOGY AND THE PRACTICE OF MEDICINE IN THE MEDICAL INSTITUTE OF LOUISVILLE:

LUNSFORD P. YANDELL, M.D.,

PROFESSOR OF CHEMISTRY AND PHARMACY IN THE SAME:

THOMAS W. COLESCOTT, M.D.

### TO SUBSCRIBERS.

We regret to find that, notwithstanding unusual expenditures upon the present volume of this Journal and its very material improvement, our subscribers have this year been more than usually remiss. We would give notice that all who may not remit promptly shall be forthwith and constantly harrassed with every form of dun until they are worried out. Their names shall appear in a black list on the back of the Journal, and, simultaneously, they shall be called upon and persecuted by duns of every kind, travelling agents, local agents, constables, sheriffs, and catchpoles. We are shamefully deprived of our dues and we mean to have justice done us.

☞Receipt by mail, at our risk, postpaid.

PRENTICE & WEISSINGER.

### PROSPECTUS OF THE SECOND SERIES

OF THE

### *Receipts of Medical Journal for April*, 1846.

| | |
|---|---|
| Dr. D. C. Ross, Foster's P. O., Ark. | $10 00 |
| Dr. T. Brown, Brushy Fork, Ky., | 5 00 |
| Dr. J. P. Smith, Starkville, Miss., | 5 00 |
| Dr. R. H. Scott, Pine Bluff, Ark., | 2 50 |
| Dr. J. A. Cotton, Edward's Depot, Miss., | 5 00 |
| Dr. Heady, Taylorsville, Ky. | 5 00 |
| Dr. W. H. Cocke, Oakland, Miss., | 5 00 |
| Dr. W. E. McCarley, Prairie Point, Miss., | 5 00 |
| Dr. D. Wilson, Hebron, O., | 5 00 |
| Dr. W. S. Rigeley, Cincinnati, O., | 5 00 |
| Dr. L. Rigdon, Hamilton. O., | 5 00 |
| Dr. R. Thompson, Columbus, O. | 10 00 |
| Dr. J. M. Bigelow, Lancaster, O., | 10 00 |
| Dr. J. W. McQuinn, Bainbridge O., | 5 00 |
| Dr. C. A. Trimble, Chilicothe, O., | 5 00 |
| Dr. E. H. Davis, Chilicothe, O., | 10 00 |
| Dr. R S. Newton, Gallapolis, O., | 5 00 |
| Dr. J. Martin, Xenia, O., | 5 00 |
| Dr. J. Pritchitt, Centreville, Ind., | 5 00 |

---

The WESTERN JOURNAL OF MEDICINE and SURGERY is published monthly by the undersigned, at the corner of Main and Fifth streets, Louisville, at $5 per annum, payable in advance. Each number contains 96 pages, making two volumes in the year of more than 550 pages.

Contributions to its pages by the Physicians of the Valley of the Mississippi, addressed to the Editors, are respectfully solicited.

Letters on business, to be addressed, postage paid, to the publishers.

*January*, 1844. PRENTICE & WEISSINGER.

